# Large Pericardial Cyst Mimicking Recurrent Unilateral Pleural Effusion on CT Scan: A Case Report and Literature Review

**DOI:** 10.7759/cureus.47735

**Published:** 2023-10-26

**Authors:** Anas R Al Khalifa, Ahmed Al Khalifa

**Affiliations:** 1 Thoracic Surgery, King Fahad Specialist Hospital, Buraiydah, SAU; 2 General Practice, Sulaiman Alrajhi University, Al Bukayriyah, SAU

**Keywords:** pericardial cyst management, video-assisted thoracoscopic surgery, computed tomography, pleural effusion, pericardial cysts

## Abstract

Pericardial cysts are an uncommon, benign condition that can manifest with diverse clinical symptoms influenced by their size and position within the body. Detecting pericardial cysts typically relies on imaging studies for a conclusive diagnosis. Surgical removal remains the definitive treatment approach for addressing pericardial cysts. This case report presents the clinical course of a 56-year-old female with a known case of asthma, and rheumatoid arthritis (RA) which exhibited recurrent symptoms such as shortness of breath and cough with recurrent pleural effusion to be investigated for suspected empyema, encysted effusion, TB, or malignancy, leading to diagnostic challenges. Through a combination of reviewing the case's clinical history, imaging modalities, and diagnostic procedures, including serial computed tomography (CT) and x-rays, the accurate diagnosis of a pericardial cyst sized 4.4 cm x 10.5 cm x 6.2 cm was achieved. In this specific case, recurrent percutaneous pleural aspirations were attempted as a treatment approach for three years. However, despite these efforts, this method proved unsuccessful in effectively managing the patient's condition. Using minimally invasive techniques, video-assisted thoracoscopic surgery (VATS) proved valuable in providing effective diagnostic and therapeutic options with reduced invasiveness. Timely diagnosis, proper monitoring, and patient education contributed to the patient's overall recovery.

## Introduction

Pericardial cysts (PCs) are rare intrathoracic lesions that arise from congenital causes or other etiologies such as inflammation, trauma, or complications of cardiac surgery [1]. They have varying clinical presentations and are found in approximately one out of every 100,000 people [2]. These cysts are typically benign and are characterized by fluid-filled sacs lined with mesothelial cells surrounded by connective tissue. PCs are uncommon intrathoracic structures typically discovered incidentally. They often arise from incomplete fusion during embryogenesis, resulting in herniation or weakness in the pericardial sac and diverticulum formation [2,3]. The relationship between the pericardial diverticulum and cyst, originating from the same embryonic source, was first recognized by Rohn A in 1903 [3]. It can also develop as acquired conditions secondary to pericarditis, inflammatory disorders, or previous surgeries. PCs typically grow in the right cardiophrenic triangle (51%-70%), with the left cardiophrenic triangle (28%-38%) and other areas of the mediastinum being less common locations [4].

Most PCs, accounting for over 50%, are asymptomatic and are commonly discovered incidentally during medical evaluations or imaging studies [5]. However, in some cases, PCs can cause symptoms such as dyspnea, chest pain, or persistent cough. Uncommon presentations include hemoptysis, fever, and pneumothorax [6]. The clinical manifestation and potential complications depend on factors such as the cyst's size, location, and whether it affects nearby structures. PCs are more commonly observed in middle-aged adults, typically occurring in the third or fourth decade of life. The prevalence of PCs shows no significant gender predilection, affecting men and women equally [7].

We present a fascinating case of a female with a large PC situated in an unusual location, mimicking the appearance of a pleural effusion on the left side. This case report aims to present a successful surgical excision of a giant PC.

## Case presentation

**Figure 1:**
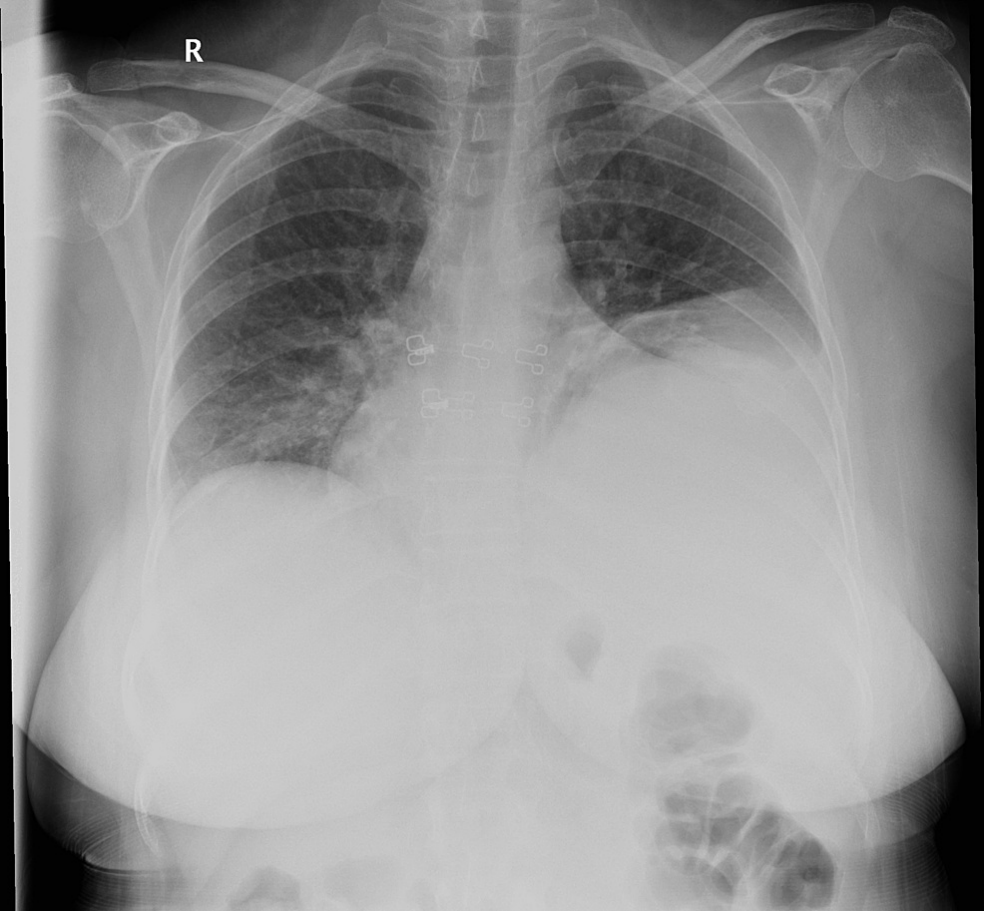
The chest x-ray displayed an opacity extending into the left costophrenic angle.

A 56-year-old female presented to King Fahad Specialist Hospital (KFSH)-Buraidah in January 2019 with complaints of shortness of breath (SOB) and persistent productive cough with white sputum for one week. High-resolution computed tomography (HRCT) of the chest reported the presence of a large left encysted pleural effusion. Previous medical reports in other hospitals indicated that she had experienced recurrent pleural effusion for five years without a definitive diagnosis. A thoracentesis of 500 mL was performed to tap the pleuritic fluid; the results were negative for acid-fast bacilli, showing moderate lymphocytes. The effusion was considered to be chronic parapneumonic. Pneumology OPD then followed the patient as a case of parapneumonic encysted pleural effusion and as a known case of asthma. The following month, a CT chest showed a mild pleural effusion on the left hemithorax, within the left central fissure and surrounding the left lingula. After three months, the patient was referred from the IM clinic to the rheumatology clinic. The patient respiratory symptoms improved, but she complained of bilateral hand stiffness and pain for five months, increasing in intensity and morning stiffness, and was diagnosed with seronegative RA. After six months, the patient returned with complaints of left pleuritic chest pain, aggravated by the right lateral decubitus position, and persistent SOB. An x-ray showed a suspected mild pleural effusion with obliteration of the left costophrenic angle (Figure 1). Moreover, she was prescribed Symbicort, Ventolin, and methotrexate medications, and the patient was followed in OPD, with instructions to return to the hospital if symptoms did not improve.

In January 2021, the patient revisited the hospital, reporting frequent coughing worsening with winter, dust, and fumes. HRCT reports concluded a moderate to marked pleural effusion within the left hemithorax that had undergone aspiration, and the symptoms resolved. Again, after seven months, aspiration was repeated, and follow-up CT reported moderate left pleural effusion with underlying lung collapse, which had decreased in amount compared to the previous study.

The following year (December 2022), the patient visited the pneumology OPD hospital complaining of the same recurrent respiratory symptoms. Her CT report traced the condition as a moderate left pleural effusion with underlying lung collapse, which was misled to be an encysted or free pleural effusion, sometimes disappearing with complete evacuation and reappearing again.

**Figure 2:**
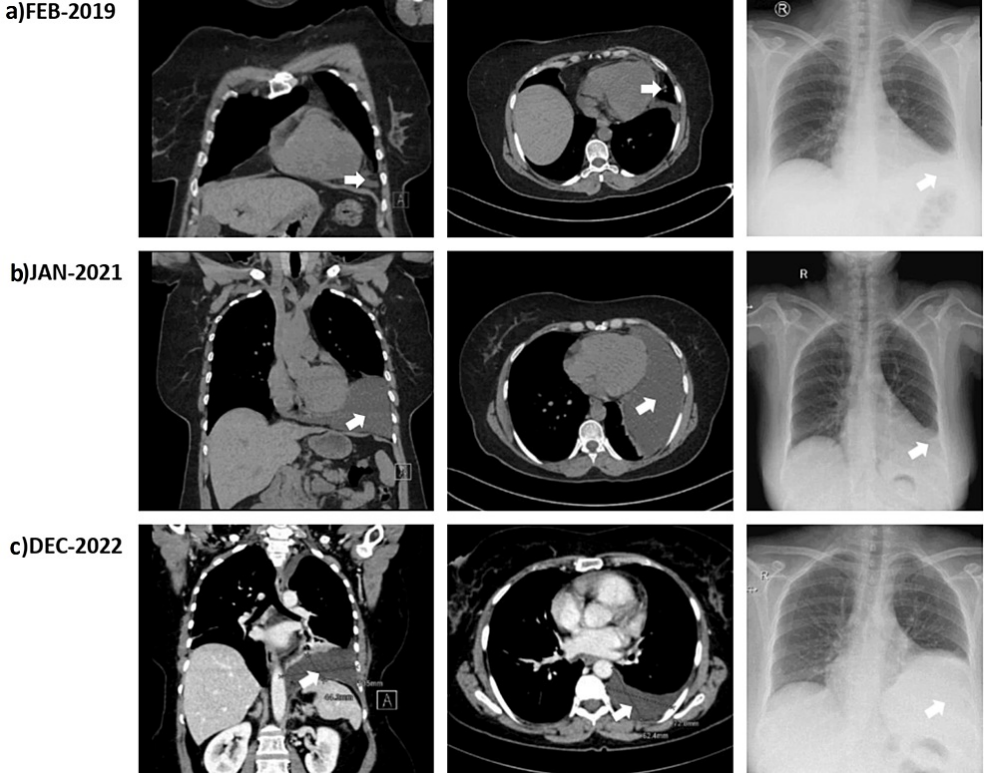
Serial CT scans and chest x-ray findings of left-sided unilateral low attenuating opacity changing size and shape, mimicking the appearance of a pleural effusion. CT: computed tomography The left column shows the coronal view of the CT The middle column shows the transverse view of the CT The right column shows the x-ray images (a) February 2019: CT chest done without contrast, (b) January 2021: CT chest done without contrast, (c) December 2022: CT chest and abdomen w/contrast.

**Figure 3:**
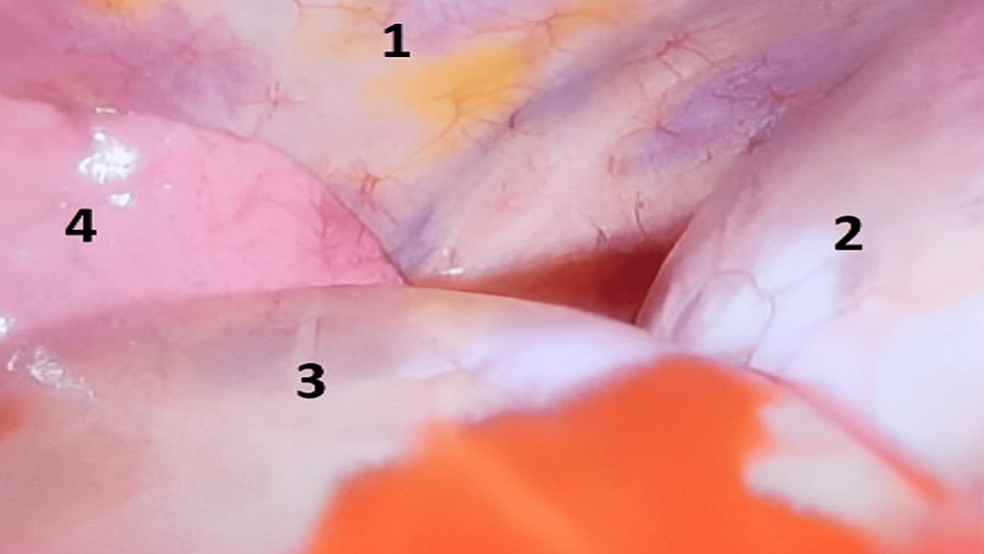
Video-assisted thoracoscopic surgery (VATS) revealing a sizeable pericardial cyst in the left cardiogenic angle, compressing the left lower lobe and the lingula. (1) Lateral chest wall, (2) Left dome of the diaphragm, (3) pericardial cyst, and (4) left lung.

**Figure 4:**
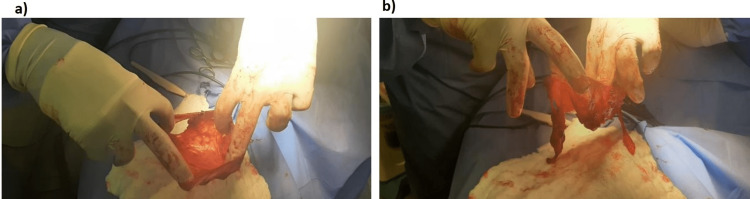
Intraoperative images showing the pericardial cyst attached to a peduncle. (a) The cyst before excision of the peduncle. (b) After the excision of the peduncle.

**Figure 5:**
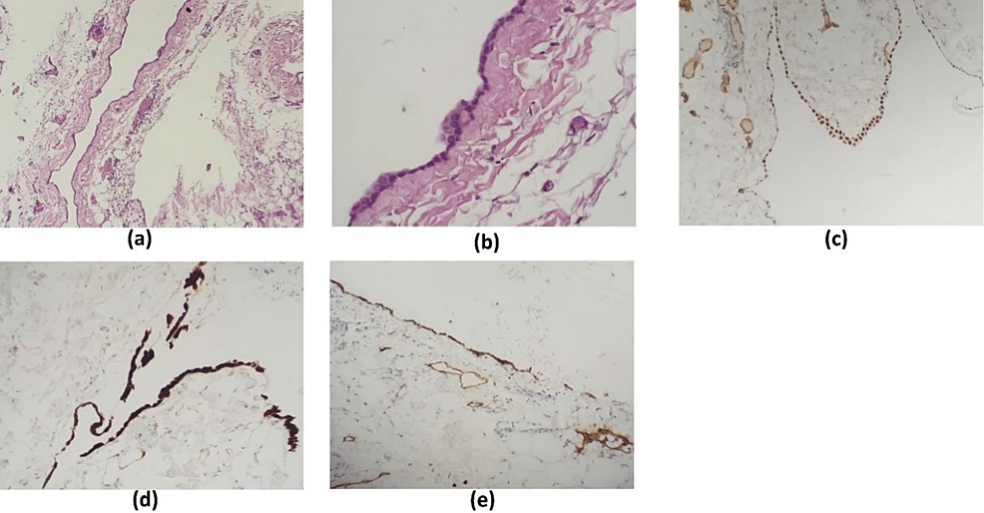
Histopathologic examination confirmed pericardial cyst diagnosis with collagen, elastic fibers, thin mesothelial lining, and chronic inflammation. (a) Cyst wall lined by bland mesothelial cells, (b) high-power view of mesothelial cells, (c) WT1 positive stain of mesothelial cells, (d) calretinin positive, (e) D2-40 positive.

At this stage, the case was referred to the thoracic surgery department as a recurrent pleural effusion for pleural biopsy to confirm the diagnosis. Considering the clinical history of the recurrence of the effusion, the nature of the aspirated fluid (clear and watery), echocardiography imaging, and Serial CT results, which showed changes in shape, size, and position over time (Figures 2a-2c), a provisional diagnosis of the PC was considered. Thus, video-assisted thoracoscopic surgery (VATS) was adopted and performed, revealing a sizeable PC (Figure 3) situated in the left cardiogenic angle compressing the left lower lobe and the lingula; it was free on the left-sided parietal pleura and the diaphragm. However, due to extensive adhesions to the lingula of the lung and the anterior surface of the pericardium, a mini left-sided thoracotomy was performed after releasing the cyst margins and adhesions, The PC became released and only was attached to a peduncle which was held with forceps and excised (Figures 4a, 4b), with no other abnormalities found. A histopathology specimen was sent for diagnosis (Figures 5a-5e); a chest tube was inserted.

**Figure 6:**
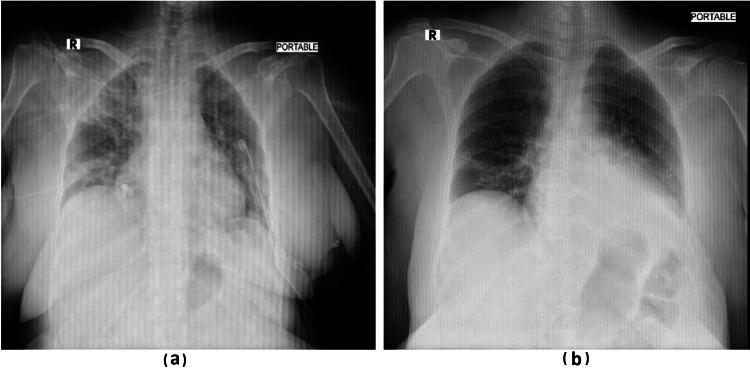
Postoperative chest x-ray showed no complications, and after the third postoperative day, the chest tube was removed. (a) Chest x-ray before chest tube removal and (b) chest x-ray after chest tube removal.

**Figure 7:**
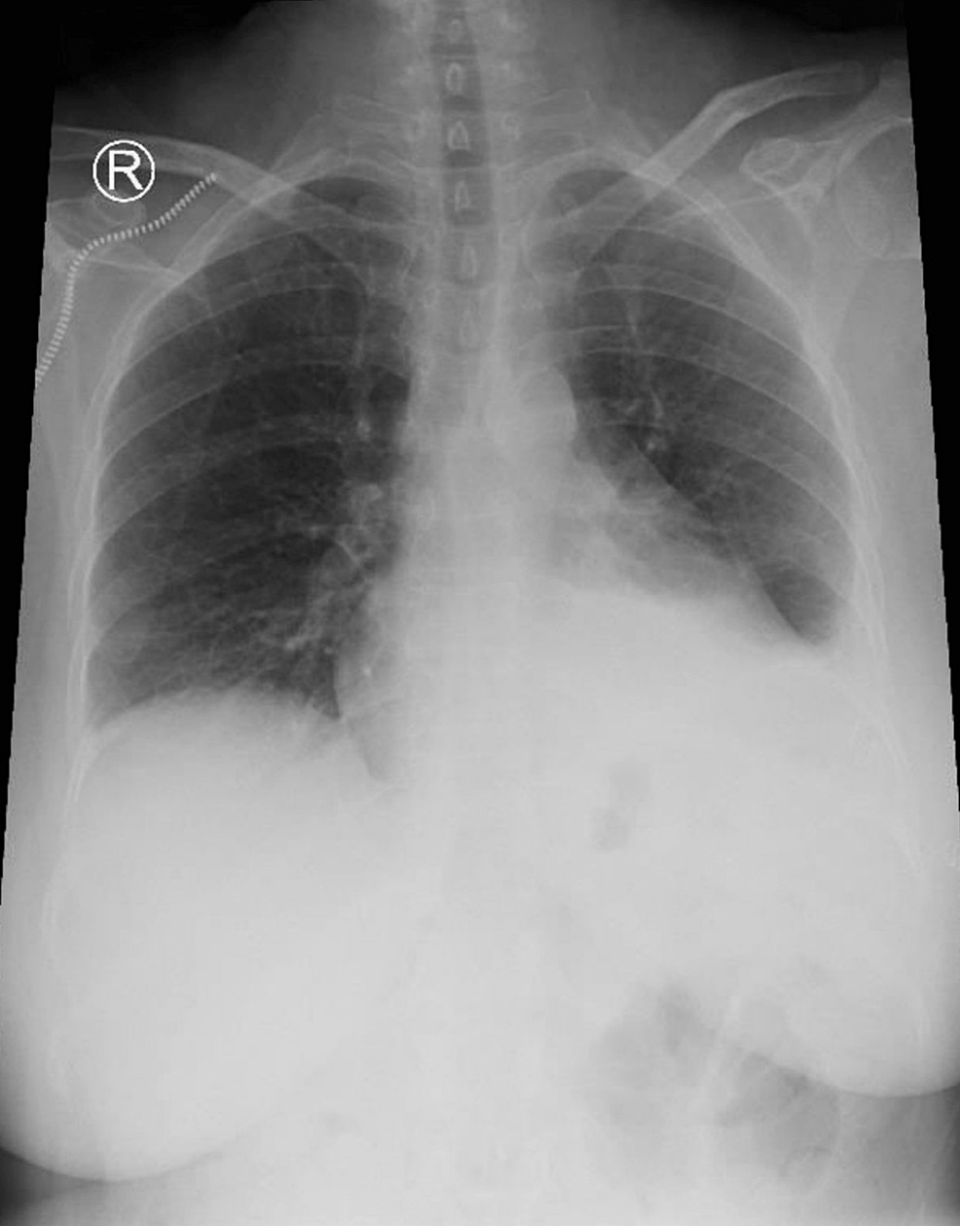
Chest x-ray showed the patient's complete recovery, after seven months of follow-up.

A postoperative x-ray was done (Figure 6a). The patient did not experience any complications, and on the third day post-op, the chest tube was removed, and the patient was discharged home (Figure 6b). Subsequent follow-ups showed no recurrence clinically or on imaging. After seven months of follow-up, the patient had no recurrence (Figure 7).

VATS is a minimally invasive surgical technique used in thoracic procedures that eliminates the need for a formal thoracotomy incision [2]. Despite the restricted access to the thorax, VATS still provides adequate visualization, making it a viable option for patients with debilitated health or marginal pulmonary reserve. This less invasive approach offers an effective alternative for performing thoracic surgeries, reducing surgical trauma and potential complications, particularly in high-risk patients [2,8].

## Discussion

PCs, which occur in approximately one person per 100,000 and comprise 7% of all mediastinal tumors, are benign intrathoracic lesions [6]. These cysts are typically found at the right cardiophrenic angle (51%-70%) or left cardiophrenic angle (28%-38%), with rare occurrences in other non-adjacent mediastinal locations (8%-11%) [4]. Histologically, they are lined with a single layer of mesothelial cells and surrounded by connective tissue containing collagen and elastic fibers. Due to their clear fluid content, they are often called “spring water cysts” [6]. Cysts typically exhibit a characteristic radiographic appearance, appearing as well-defined, round, or oval masses with smooth walls. They can be either unilocular or multilocular and have a diameter ranging from 1 to 5 cm [9]. The diameter of the PC found in this study was also 4.2 cm.

The age distribution of PCs needs to be better defined. While there have been reported cases of PCs in individuals younger than eighteen, the literature predominantly consists of reports involving older patients; our patient said their age was 56. There are documented cases of PCs in various age groups, but the overall distribution across different age ranges remains to be determined [5].

PCs commonly arise from congenital causes resulting from the incomplete fusion of mesenchymal lacunae during embryogenesis [10]. This developmental anomaly is considered the primary etiology for PCs. However, the literature also describes other potential causes of PCs. Inflammation can play a role in their formation, such as in cases of rheumatic pericarditis, bacterial infections (especially tuberculosis), and echinococcosis [7]. Additionally, PCs can develop due to trauma, complications arising from cardiac surgery, or as a complication of chronic hemodialysis [8]. These various etiologies highlight that PCs can arise from different underlying factors beyond the congenital origin, and a comprehensive evaluation is necessary to determine the specific cause in each case. While PCs are usually discovered incidentally on chest x-rays in asymptomatic individuals (50%-75%), they can cause symptoms when they exert pressure on nearby organs [2]. In this reported case, the patient suffered from cough, SOB, and chest pain. Feigin et al. reported symptoms such as atypical chest pain, dyspnea, and persistent cough in approximately one-third of patients [11].

Diagnosing PCs can be particularly challenging due to their infrequent occurrence, mainly when located outside the typical cardiophrenic angles [6]. Also, the diagnosis becomes more complicated when the cyst involves mediastinal and thoracic structures, leading to an atypical presentation [12]. PCs are commonly detected and monitored using radiological modalities. They are often discovered as incidental findings during asymptomatic individuals' chest x-ray scans. Transthoracic echocardiography is particularly useful in providing precise information about the location and characteristics of the cyst, leading to improved recognition of the lesion. Magnetic resonance imaging (MRI) and computed tomography (CT) are valuable imaging techniques for PCs, offering detailed information about the mass's density, the cyst's anatomical location, and its relationship with surrounding structures [13]. Diagnostic procedures used in this case report were x-rays, CT scans, and through histopathological examination. These imaging modalities also aid in differentiating PCs from other abnormalities, such as thoracic malignancies. Therefore, histological examination through biopsy or surgical resection is often necessary to obtain a definitive diagnosis and confirm the nature of the cyst. The histological examination provides a consolidated diagnosis by examining the tissue samples, allowing for a comprehensive assessment and accurate characterization of the PC [8].

Literature review

Nina et al. reported a 46-year-old female who presented with symptoms of mediastinal compression caused by a giant PC on the right side. The results of an echocardiogram initially suggested the diagnosis of a PC and were later confirmed by both a thoracic CT scan and nuclear magnetic resonance imaging. The imaging studies revealed a cyst measuring 13 x 9.5 cm. Surgical cyst excision was performed, leading to complete remission of symptoms during a 12-month follow-up period [14].

This report was presented by Kaklikkaya, a case of a giant PC in a 39-year-old male. The cyst, measuring 22 × 15 × 7 cm, exerted pressure on the heart and lungs. Surgical excision of the cyst was performed through left-side thoracotomy. Following the procedure, the patient has remained asymptomatic for about nine years and appears to be in complete remission [1].

Simsek et al. reported a case involving a 28-year-old man initially presenting with acute bronchitis. However, a chest x-ray led to a misdiagnosis of dextrocardia. Further evaluation with thorax CT revealed a large, well-defined mass measuring 6.4x9 cm in the middle mediastinum, suggesting a PC. Serial imaging studies showed no change in the size of the cyst over time, and the patient remained utterly asymptomatic during a 12-month follow-up period. As a result, surgical resection of the cyst was not executed, and conservative management was adopted [15].

Hekmat et al. presented a case of a 24-year-old man who contributed to the emergency department with dyspnea and a persistent cough. Trans-thoracic echocardiogram showed an echo-lucent space adjacent to the right atrium at the right cardiophrenic angle, without any pericardial effusion. The patient subsequently underwent surgery, during which a large cyst measuring approximately 13 × 8 × 5 cm was found outside the pericardium on the right side. The cyst was excised entirely, and the patient was discharged after five days. The pathologic report confirmed the preoperative diagnosis of a PC [5].

In a case by Makar et al., a unique presentation of a large PC was observed in a 43-year-old female patient. The patient presented with breathlessness and pleuritic chest pain, leading to the discovery of a large PC located adjacent to the right atrium. A follow-up CT scan showed a significant mass on the right side, extending into the right chest, measuring 5.1 cm × 9 cm × 4.3 cm. Due to the rapidly worsening symptoms, surgical resection was performed using VATS. The patient suffered the procedure well and experienced no postoperative complications [16].

In a case study conducted by Li et al., a rare case of a large PC in a 36-year-old woman was reported. The cyst was located in an uncommon location, mimicking a unilateral pleural effusion. The diagnosis was confirmed, and resolution was achieved through VATS. During the VATS procedure, it was discovered that the previously presumed loculated effusion was, in fact, a PC. The cyst, filled with brown fluid, measured 10.5 cm × 2.5 cm × 2 cm in size. It originated from the lateral aspect of the pericardium near the left ventricle and was adhered to the lung and pleura. After successfully removing the PC, the patient's symptoms improved. A follow-up chest CT performed six months after discharge showed no evidence of recurrence. Based on this case, the study suggests that VATS is a feasible and safe method for treating symptomatic and large PCs [8].

**Table 1:** Clinical presentations and diagnostic challenges of pericardial cysts: a summarized literature review

References	Age	Gender	Size (cm)	Location	Treatment
Nina et al. [14]	44	Female	13 × 9.5	Right side	Right side thoracotomy
Kaklikkaya [1]	39	Male	22 × 15 × 7	Left side	Left side thoracotomy
Simsek et al. [15]	28	Male	6.4 × 9	Right side	Conservative management
Hekmat et al. [5]	24	Male	13 × 8 × 5	Right side	Mid sternotomy
Makar et al. [16]	43	Female	5.1 cm × 9 cm × 4.3	Right side	Video-assisted thoracoscopic surgery
Li et al. [8]	36	Female	10.5 cm × 2.5 cm × 2	Left side	Video-assisted thoracoscopic surgery

Although PCs typically have a benign course, complications have been reported, including cyst rupture, erosion into adjacent structures like the right ventricular wall or superior vena cava, cardiac tamponade, mitral valve prolapse, obstruction of the right mainstem bronchus, atrial fibrillation, and even sudden death [6]. Surgical excision is generally recommended for symptomatic patients, while asymptomatic cases are managed conservatively with close follow-up. Minimally invasive thoracoscopic resection is a favorable alternative to open surgical resection, as it reduces surgical trauma, postoperative pain, and recovery time, resulting in improved cosmetic outcomes [2]. Percutaneous aspiration of cyst contents may be another appealing alternative to surgical resection for only symptomatic relief three-year follow-up study of our patient undergoing aspiration multiple times. In rare cases, spontaneous resolution of PCs has been reported, possibly due to cyst rupture.

The management approach for PCs depends on several factors, including the presence of symptoms, complications, and the size and location of the cyst. In many patients, close monitoring with serial transthoracic echocardiography is sufficient to ensure a benign course and allow for potential spontaneous resolution of the PC. If intervention is necessary, treatment options include percutaneous aspiration, surgical intervention using VATS, or surgical excision [8-10,14]. Percutaneous aspiration is a minimally invasive approach that can be attempted for diagnostic purposes and therapeutic relief of symptoms. However, in some cases, it may not yield definitive results or provide a specific diagnosis. In situations where PCs cause significant symptoms or have cardiorespiratory repercussions, surgical intervention becomes necessary. Due to the recurrence of the symptoms, the patient in this report decided to be treated with VATS and mini left thoracotomy [17]. VATS, a minimally invasive surgical technique, is viable and promising for removing PCs in symptomatic patients [8]. In cases where the PC was large enough to cause symptoms, surgical intervention was deemed necessary. The overall morbidity and mortality associated with managing OCs are low. Surgery, specifically cyst resection, has been demonstrated as the only definitive curative treatment for PCs [5].

## Conclusions

We presented a rare case involving a large PC in an uncommon site (left side). In the described case, the presentation mimicked a recurrent free unilateral pleural effusion, and thus, many attempts were made to perform percutaneous aspiration for further diagnostic workup, but this approach was unsuccessful. The definitive diagnosis was ultimately established through VATS and the process of mini-thoracotomy, which through the PC, was successfully removed. Therefore, VATS played a pivotal role in effectively establishing a precise diagnosis of the PC achieving a favorable outcome for the patient. This less invasive approach reduced surgical trauma, improved postoperative recovery, and proved especially beneficial for a patient with marginal pulmonary reserve.
